# A Central Asian Food Dataset for Personalized Dietary Interventions

**DOI:** 10.3390/nu15071728

**Published:** 2023-03-31

**Authors:** Aknur Karabay, Arman Bolatov, Huseyin Atakan Varol, Mei-Yen Chan

**Affiliations:** 1 Institute of Smart Systems and Artificial Intelligence, Nazarbayev University, Astana 010000, Kazakhstan; 2 School of Medicine, Nazarbayev University, Astana 010000, Kazakhstan

**Keywords:** nutritional intervention, computer vision, food classification, Central Asian food, dietary assessment, food recognition, AI, Central Asia, food dataset

## Abstract

Nowadays, it is common for people to take photographs of every beverage, snack, or meal they eat and then post these photographs on social media platforms. Leveraging these social trends, real-time food recognition and reliable classification of these captured food images can potentially help replace some of the tedious recording and coding of food diaries to enable personalized dietary interventions. Although Central Asian cuisine is culturally and historically distinct, there has been little published data on the food and dietary habits of people in this region. To fill this gap, we aim to create a reliable dataset of regional foods that is easily accessible to both public consumers and researchers. To the best of our knowledge, this is the first work on the creation of a Central Asian Food Dataset (CAFD). The final dataset contains 42 food categories and over 16,000 images of national dishes unique to this region. We achieved a classification accuracy of 88.70% (42 classes) on the CAFD using the ResNet152 neural network model. The food recognition models trained on the CAFD demonstrate the effectiveness and high accuracy of computer vision for dietary assessment.

## 1. Introduction

Recent developments in “omics” technology have made it tempting to collect and bank large amounts of biological material. One subfield of this area, foodomics, has recently attracted the interest of researchers thanks to its potential to expand our understanding of the biochemical profile of food and its effects on physiological processes in our bodies [1]. However, if the measurement of dietary and lifestyle factors is ignored or collected with inappropriate instruments, this would potentially diminish the expected health benefits of the genome and large-scale cohort studies [2]. This might also diminish the full potential of precision lifestyle medicine and the application of positive dietary and lifestyle interventions. Therefore, it is vital to give due attention to appropriate assessment of dietary intake [3,4]. Current methods for assessing dietary intake are cumbersome and generate data that require a great deal of effort to code and subsequently analyze. Another limitation of using traditional approaches is the subjective and inconsistent classification of food groups by different individuals. In recent decades, artificial intelligence (AI) has begun to penetrate the food industry by offering promising approaches to the modeling and improvement of food product characteristics [5], recipe evaluation [6], food identification, and dietary analysis [7].

In recent years, food computation from visual data has become a prominent area of research thanks to computer vision (CV) development and the increasing use of smartphones and social media [8]. These platforms have enabled access to a wide range of food-related information, including images, recipes, and consumption logs. As a result, these data can be used for various tasks, from influencing our behavior and culture to improving medical, biological, gastronomic, and agronomic research. At the forefront of these efforts is the development of deep learning-based food image recognition systems with multiple applications in dietary assessment, smart restaurants and supermarkets, food safety inspection and control, and agriculture. Automatic food image recognition and classification can increase the accuracy of nutritional records in various devices (e.g., smartphones) and offers considerable benefits in assisting visually impaired people [8]. A number of datasets have been collected for food classification, localization, real-time recognition, and quantity evaluation [9,10].

Most of the existing food classification datasets are web-crawled collections and include Western, European, Chinese, and other Asian cuisines (see Table 1). For example, Bossard et al. [11] created the Food-101 dataset, which contains 101 European food classes with 1000 images per class and has become a benchmark for many recognition models and datasets [12,13]. The fine-grained Chinese food dataset VireoFood-172 [14] and its follow-up Vireo-Food251 [15] have been employed for ingredient recognition systems [15]. Another large-scale dataset ISIA Food-500 was introduced by Min et al. [16] and contained 500 categories with over 400,000 images. The dataset contains Asian, European, and African food. Sahoo et al. [10] developed a food recognition system called FoodAI that uses deep learning and can be deployed on smartphones. FoodAI was trained on a dataset of 400,000 images from the Internet and can recognize 756 food classes, mainly foods eaten in Singapore [10]. To date, the most comprehensive large-scale dataset is Food2K [17]. This dataset contains over one million images across 2000 food classes from different cuisines. The dataset is fine-grained, meaning that various classes for the same food type differ in ingredients. The two largest food datasets, FoodAI and Food2K, can significantly enhance food computation models. However, FoodAI is not open source, while Food2K is not publicly available. Nevertheless, the developers of the Food2K dataset have released a food recognition challenge dataset called Food1K, which contains approximately 400,000 images, and as the name implies, 1000 food classes [18].

As mentioned earlier, most food datasets contain predominantly Western and Asian dishes consumed around the world, rather than specific national dishes such as those found in Central Asia. To create a system capable of recognizing food specific to a certain region, local preferences, specialties, and cuisines should be considered. For example, ref. [18] introduced the Turkish Food Dataset, which contains 15 Turkish food items. Therefore, we aimed to develop and create a unique food recognition system specific to our region that takes into account the way food is prepared, served, and consumed, as well as other local preferences.

The datasets listed in Table 1 paved the way for the development of food recognition models. For instance, Aktı et al. [19] developed a mobile food recognition system that achieved an accuracy of 94% on 23 Middle Eastern food items. Another study addressed the integration of convolutional neural networks (CNNs) and text models to predict and analyze the nutrient content of food images and food ingredients [20]. Based on the MyFoodRepo dataset, which contains 24,119 images and 39,325 polygons (i.e., the number of food items), an instance segmentation model was proposed in [21]). The authors experimented with different models to show that the precision in predicting the food ingredients can be increased.

Central Asia has one of the highest rates of premature mortality from non-communicable diseases (NCDs), such as cardiovascular diseases, diabetes, and certain types of cancer [22]. Dietary habits are one of the major factors contributing to the prevalence of NCDs. In fact, a recent study of about 200 countries showed that the burden of diet-related deaths in Central Asia is among the highest in the world [23]. The resulting premature deaths and illnesses negatively impact socioeconomic development and undermine progress toward sustainable development goals (SDGs) [24].

Investigating the associations between dietary intake and other lifestyle factors with cardio-metabolic risk factors in adult Central Asians would provide evidence for public health policy. In addition, integrating AI into smartphone diet-tracking applications could significantly improve nutrition literacy among local populations. Since AI requires data to create models, this work introduces the first dataset of Central Asian food images and deep learning-based food classification models trained on these data. The Central Asian Food Dataset (CAFD) contains more than 16,000 images of 42 national and local foods not included in any of the datasets listed in Table 1. We performed extensive parametric experiments to illustrate the performance of the models trained on the CAFD. Additional experiments were conducted to build food recognition models using the combined CAFD and Food1K datasets, which is currently one of the largest datasets in terms of the number of classes. Furthermore, this work will help to facilitate future nutrition research to be conducted in this field for these ethnic populations.

The remainder of the paper is as follows: Section 2 presents the methods used to develop the CAFD, specifically, data collection, labeling, and other pre-processing steps. Section 3 explains the food recognition models and details the parametric experiments. Section 4 discusses the food recognition model performance, and Section 5 concludes the paper.

## 2. Central Asian Food Dataset

**Figure 1:**
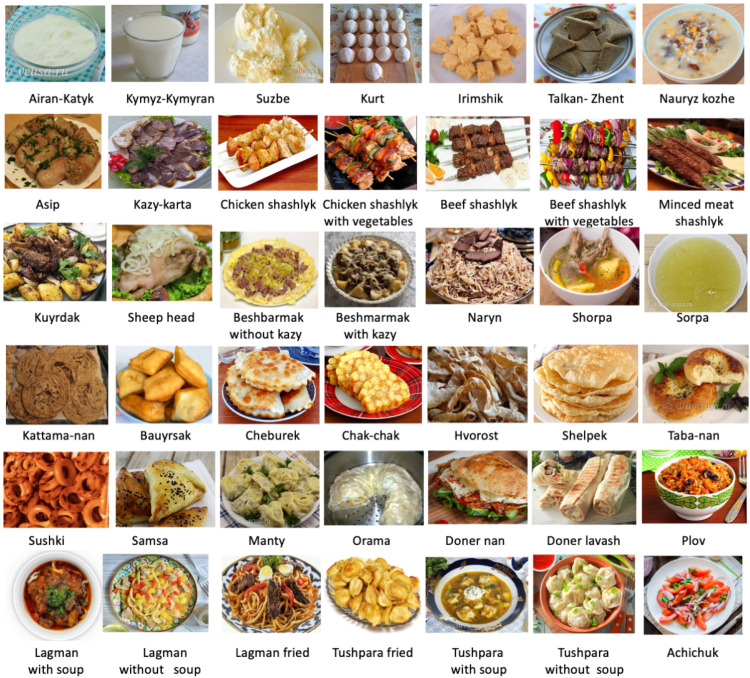
Sample images for Central Asian Food Dataset classes.

In this paper, we present a novel large-scale Central Asian Food Dataset (CAFD) (see Figure 1). This dataset is composed of 16,499 images with 42 classes encompassing the most popular Central Asian cuisine consumed locally. We conducted extensive data cleaning, iterative annotation, and multiple inspections to ensure the high quality of the dataset. We envision that this large-scale, high-quality dataset could be useful for developing food image representation learning for food-related vision tasks. In addition, the CAFD can serve as a sizable fine-grained benchmark for visual recognition.

**Figure 2:**
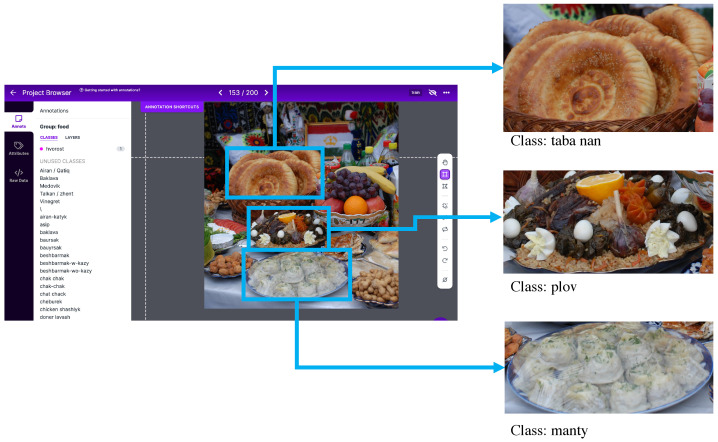
Data pre-processing: Food labeling and cropping on Roboflow. Original image and final cropped images with the respective labels.

To obtain a high-quality food image dataset with broad coverage, high diversity, and high sample density, we followed a five-step process. First, we created a list of the most popular food items eaten in Central Asia. Second, we scraped images from popular search engines (e.g., Bing, Google, YouTube, and Yandex) and social media websites (e.g., Instagram and Facebook) using query words in different languages. We wrote a Python script using the Selenium library to automatically download images from the Internet. To increase the number of images in the underrepresented classes (e.g., sheep head, asip, and nauryz-kozhe), we scraped recipe videos from YouTube, cropped parts with the finished dish, and extracted certain frames. Images from the videos were automatically extracted using the Roboflow [25] software at a rate of one frame per second to obtain food images from different camera angles and under different lighting conditions. To ensure the high quality of the dataset the HashImage Python library was used to conduct exact duplicate removal. Most of the images contained multiple food items and background clutter. Since this work focuses on food image classification, we needed a single food item per image. Therefore, in the third step, two image annotators created bounding boxes for each food item in the images using the Roboflow software Figure 2. Each bounding box has a label (i.e., 0 to 41 for the 42 classes) indicating the food item contained within.

Fourth, we extracted all of the images and their label files from Roboflow. Each image has its respective label file in the “.txt” format that contains the coordinates of the bounding box and its class. Next, we cropped the food items from the original images based on their bounding box coordinates, as shown in Figure 2. The final images were stored in separate directories based on the food class. Sample images for the 42 classes are shown in Figure 1. All images in this paper are from Wikipedia and delo-vcusa.ru and are provided under the Creative Commons (CC) license (creativecommons.org/licenses/by-nc-nd/4.0/ (accessed on 15 February 2023)).

**Figure 3:**
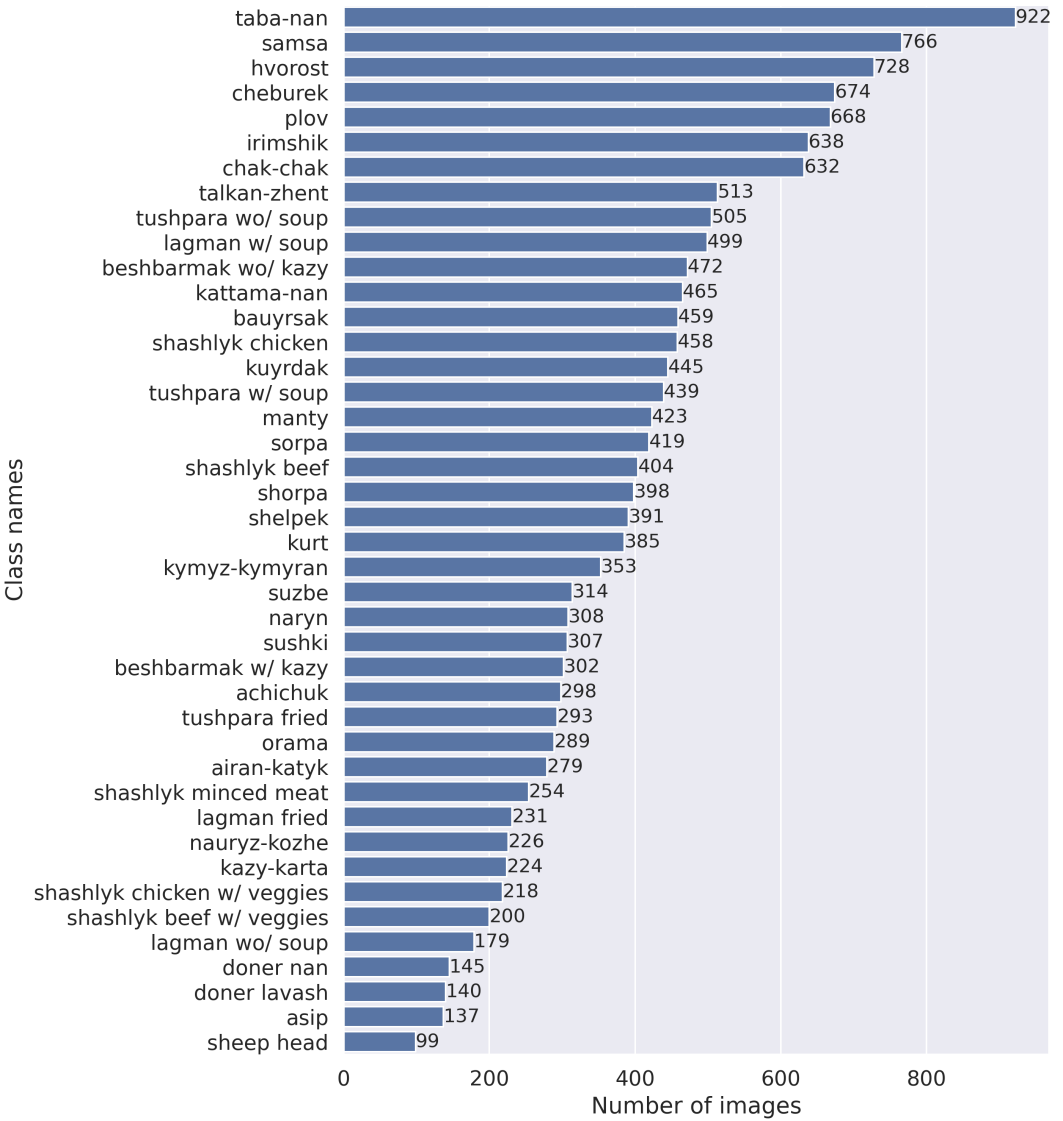
CAFD statistics by class.

The final dataset has an imbalanced number of images per class, ranging from 99 to 922. Figure 3 illustrates the distribution of images per class. The dataset is publicly available in our GitHub (https://github.com/IS2AI/Central-Asian-Food-Dataset (accessed on 23 March2023)) repository.

## 3. Food Recognition Models

Image classification is a computer vision task that extracts a single descriptor (i.e., class) from an entire image. State-of-the-art image classification models are based on CNNs, which essentially employ convolutional filters to generate features from the image to identify an object. Image classification models have improved dramatically over the last decade thanks to the availability of large datasets. Indeed, training these models requires a vast amount of training data depending on the number of classes and the domain. Since it is not always feasible to collect and label a sufficient amount of training data, transfer learning is often used. Transfer learning is a technique in which some parts of a machine learning (ML) model used to solve one problem are used in solving a similar problem but in a different domain [26]. For example, transfer learning could be applied to solve the problem of classifying whether an image contains food by using the knowledge of the model obtained during training to detect whether there are any beverages in the image.

In this work, we applied transfer learning to our food classification problem using model weights pre-trained on ImageNet, a large dataset containing over 14 million images [27]. ImageNet contains 1000 different object classes (e.g., animals, technology, everyday items, plants, etc.). Classification models identify the object based on the extracted features, such as shape, color, and texture. Therefore, models pre-trained on a large number of images from ImageNet are powerful, as they learn to identify diverse shapes and features. In this case, one can take advantage of transfer learning to solve a problem with a much smaller dataset.

To verify our food recognition models, we trained them on the publicly available Food1K dataset. Further, we tested the combination of both CAFD and Food1K to obtain a food classifier with the largest number of food classes 1042 classes) known to us. This also allowed us to determine whether or not our CAFD had overlapping classes with Food1K.

Since the Food1K dataset was released for the International Conference on Computer Vision (ICCV) Food Recognition Challenge Competition, only training and validation sets were available. Therefore, we split the validation set into two equal parts to obtain a validation set and a test set. With respect to the CAFD, we split the dataset into approximately 70% for the training set, 15% for the validation set, and 15% for the test set. About 30% of the images in the final dataset are cropped from raw images with multiple food items. Thus, to avoid the bias caused by the background of the food images in the training, validation, and testing sets, we first divided the original images into the above sets and then cropped the food items. In addition, the data were in two formats: scraped images and frames extracted from YouTube videos. Since multiple frames came from each video, we split them into training, validation, and test sets to avoid data leakage during model training. Table 2 shows the number of images in the training, validation, and test sets for three different datasets.

We performed transfer learning on Pytorch using the pre-trained models on ImageNet. We selected 10 models of different architectures, complexity, and a number of parameters to evaluate their performance on the CAFD (see Table 3). VGG-16, a large early CNN-based network with 16 layers and approximately 138 million trainable parameters [28], achieved an accuracy of 92.5% on the ImageNet dataset. Squeezenet1, in contrast, is a small model with only one million trainable parameters [29]. This allows for faster training and deployment on hardware with limited memory capacity. We experimented with five different models with the residual network (ResNet) architecture [30,31,32]. Skip connections in the ResNets enabled network depth extension and better performance. DenseNet-121 and EfficientNet-b4 have architectures similar to those of ResNets, except that they aim to reduce model complexity by introducing different scaling methods [33,34].

The training was performed on a single Tesla V100 GPU on an Nvidia DGX-2 server. Models were trained for 40 epochs with a learning rate of 0.001, batch size of 64, and a categorical cross-entropy loss. The input size of images varied (i.e., 224 × 224 for VGG-16 and ResNets, 380 × 380 for EfficientNet). Because the datasets were highly imbalanced and large, we used Top-5 accuracy in addition to Top-1 accuracy as a model evaluation metric. Top-1 accuracy is the usual metric for accuracy. With this metric, the highest probability output of the model must match the ground truth exactly. An alternative measure, Top-5 accuracy, extends this concept. The ground truth class must be one of the five most probable outputs. Further, to identify and analyze the best and worst-classified food classes, we used the precision, recall, and $F_{1}$-score metrics. Precision indicates how many of the samples in a given class (e.g., images of “samsa”) are correctly classified. Recall, on the other hand, indicates the proportion of images actually containing the food class “samsa” measured against all samples predicted as “samsa”. $F_{1}$-score is the harmonic mean of precision and recall and is computed as follows:

$$F_{1}-\mathrm{score}=\frac{2\cdot \mathrm{Precision}\cdot \mathrm{Recall}}{\mathrm{Precision}+\mathrm{Recall}}$$

## 4. Results and Discussion

The results of the classification models are summarized in Table 3. Overall, all models performed better on the CAFD than on both Food1K and CAFD+Food1K. Compared to Food1K, all models obtained slightly better results on CAFD+Food1K, indicating the accuracy and cleanness of the CAFD. Furthermore, this implies that there are no classes in CAFD and Food1K that overlap significantly.

VGG-16 achieved 86.03% for Top-1 accuracy and 98.33% for Top-5 accuracy on the CAFD. As for the Food1K and CAFD+Food1K datasets, performance was lower due to the substantially larger number of classes (1000 and 1042, respectively). Top-1 was 80.67% and Top-5 was 95.24% for Food1K, and 80.87% and 96.19% for CAFD + Food1K. The Squeezenet architecture has a smaller number of parameters, but more layers, and, unlike the VGG architecture, delays the down-sampling of the input image size toward the end of the network. Squeezenet1 achieved a Top-1 accuracy of 79.58% on the CAFD, 71.33% on Food1K, and 69.19% on CAFD + Food1K. Since the model has a small architecture, the performance decreases for larger datasets.

ResNet architectures, which can utilize very deep networks by avoiding diminishing gradients, achieved about 88% for Top-1 accuracy and approximately 98% for Top-5 accuracy on the CAFD. The Top-1 score is above 82%, and Top-5 is nearly 97% for both Food1K and CAFD+Food1K. It can be observed that as the network depth increases, the accuracy grows higher. For example, in the case of ResNet50 (50 convolutional layer blocks), the Top-1 accuracy was 88.03% and Top-5 was 98.44% for the CAFD. ResNet152, on the other hand, achieved a Top-1 accuracy of 88.70% and a Top-5 accuracy of 98.59% on the CAFD, which is the highest performance on this dataset among all models. For Food1K and CAFD+Food1K, the ResNet models showed similar performance, and the ResNet152 variant achieved the highest score within the ResNet family. Increasing the level of granularity of the captured feature by utilizing a wider network, Wide ResNet-50, improved accuracy with a Top-1 accuracy of 88.21% on the CAFD compared to ResNet50 (88.03%). EfficientNet-b4 achieved the best results on both Food1K (Top-1 is 87.47% and Top-5 is 98.04%) and CAFD + Food1K (Top-1 is 87.75% and Top-5 is 98.01%), which both had a very large number of classes in our experiments.

Table 4 and Table 5 list the 10 CAFD classes best and worst detected by the best-performing models trained on the CAFD (ResNet152) and CAFD+Food1K (EfficientNet-b4). In both cases, similar classes performed best (6 out of 10: plov, naryn, samsa, sushki, sheep head, and achichuk). Most of the best-detected classes have a high number of images or have very distinct features, shapes, or colors compared to all other classes in the dataset (see Figure 3). For example, the detection of the classes “naryn”, “plov”, and “samsa” resulted in precision scores of 96%, 93%, and 94%, respectively, (see Table 4). A precision score of 0.96 was obtained for the class “sushki” and 0.95 for “achichuk”, which have unique shapes and colors (see Table 1), indicating that almost all samples in the test set were correctly predicted. As for the worst predicted classes, 5 out of 10 classes were identical in both cases: shashlik chicken with vegetables, shashlik beef, asip, kazy-karta, and lagman without soup. These results illustrate that fine-grained or similar-looking classes cause more confusion and deteriorate the performance of the model (e.g., “shashlik chicken with vegetables” and “shashlik beef”, “kazy-karta” and “asip”). The worst scores were obtained for the food class “shashlik chicken with vegetables” (a precision score of 0.71 when trained only on the CAFD) and the class “lagman without soup” (a precision score of 0.6 when trained on CAFD+Food1K). This indicates that about 30–40% of the test samples were inaccurately predicted for these classes.

**Figure 4:**
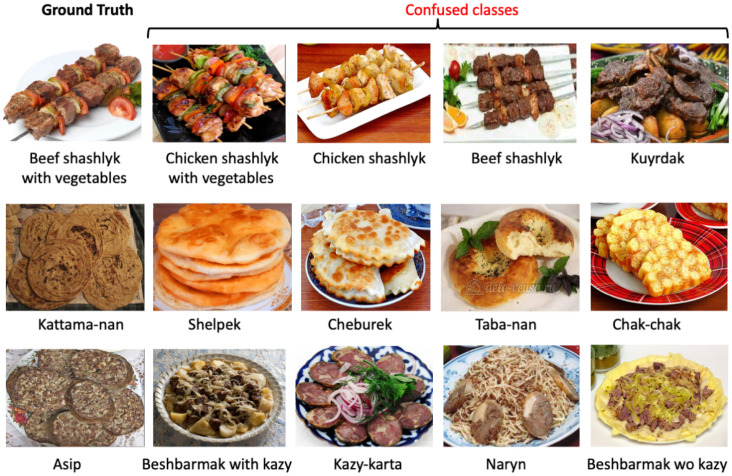
Examples of the confused classes.

Figure 4 illustrates samples of the confused classes for three cases (beef shashlik with vegetables, kattama-nan, and asip). Next to each of the (ground truth) classes are sample images of four classes that are most commonly confused with them. This suggests that further neural network topology optimization or data augmentation should be undertaken to distinguish between these food items, as the nutritional content of some of these food items differs significantly. For instance, a 100 g serving of (lean) beef shashlik provides 250 kcal, 28 g protein, and 15 g fat; chicken shashlik contains 180 kcal, 27 g protein, and 7 g fat, and mutton shashlik contains 290 kcal, 24 g protein, and 20 g fat. Therefore, for subsequent dietary analyses, there would be a difference between the fat and the total calorie intake of the individuals.

The proposed Central Asian Food Dataset has several potential applications, including the creation or modification of new recipes using ingredient combinations that are unique and commonly consumed by ethnic groups in this region. In addition, our dataset can help restaurants and food service providers plan their menus to be more appealing to target audiences in Central Asia. Food manufacturers can also use our food dataset to optimize their production processes and combat fraudulent food practices. In summary, our Central Asian Food Dataset can have a significant impact on the food industry. It can be used to improve food quality, develop new recipes and personalized dietary plans, optimize production processes, and increase food safety. Additionally, there is potential for integration with other food recognition systems.

## 5. Conclusions

With the development of CV and the availability of devices, food recognition is gaining a considerable advantage over other approaches in automating and increasing the accuracy of dietary assessment. In this work, we present the Central Asian Food Dataset, which contains 16,499 images for 42 food classes. The dataset consists of commonly consumed Central Asian dishes that are not included in the vast majority of currently existing open-source datasets. To illustrate the performance of CV models on the CAFD, we trained a number of food recognition models using this dataset. In addition, we present transfer learning results using the largest dataset currently available, CAFD+Food1K, which contains a total of 1042 classes. We have achieved a Top-5 accuracy of 98.59% and 98.01% for the CAFD and CAFD+Food1K, respectively. The source code, pre-trained models, and the CAFD are publicly available in our GitHub repository.

The performance of the food recognition models developed using the CAFD demonstrates the effectiveness and potential of our dataset for dietary analysis tools and applications. As our next step, we will explore different neural network architectures and data augmentation methods to improve the classification of some of the less accurately recognized food items. We will also explore how the CAFD can be utilized to benefit other dietary-related tasks including using it in a social media bot to capture the lifestyle and other nutritional factors of the population living in the area. In this study, we have worked with classification models for one food item per image. As a continuation of this work, we will look at food localization and create a food scene recognition dataset where multiple food items are present in a single image. To validate this dataset, we will utilize object recognition models that can locate food items in an image and classify them. It is also likely that the dataset will contain more food classes since food scenes usually include local national dishes consumed with other Western or Asian foods. Based on the additional food classes, we will be able to extend the current food categories.

## Figures and Tables

**Table 1:** Summary of food classification datasets.

Dataset	Year	# Classes	# Images	Cuisine	Public
Food-101 [11]	2014	101	101,000	European	yes
VireoFood-172 [14]	2016	172	110,241	Chinese/Asian	yes
TurkishFoods-15 [18]	2017	15	7500	Turkish	yes
FoodAI [10]	2019	756	400,000	International	no
VireoFood-251 [15]	2020	251	169,673	Chinese/Asian	yes
ISIA Food-500 [16]	2020	500	399,726	Chinese/International	yes
Food2K [17]	2021	2000	1,036,564	Chinese/International	no
Food1K [17]	2021	1000	400,000	Chinese/International	yes
**Central Asian Food Dataset (CAFD**)	**2022**	**42**	**16,499**	**Central Asian**	**yes**

**Table 2:** Image distribution across the training (train), validation (valid), and test sets.

Dataset	Train	Valid	Test
CAFD	11,008	2763	2728
Food1K	317,277	26,495	26,495
CAFD+Food1K	328,285	29,258	29,223

**Table 3:** Top-1 and Top-5 accuracies for different food classification models and datasets.

Base Model	# Parameters	CAFD		Food1k		CAFD+Food1K	
	(mln)	Top-1 Acc.	Top-5 Acc.	Top-1 Acc.	Top-5 Acc.	Top-1 Acc.	Top-5 Acc.
VGG-16 (2014) [28]	138	86.03	98.33	80.67	95.24	80.87	96.19
Squeezenet1_0 (2014) [29]	1	79.58	97.29	71.33	91.23	69.16	90.15
ResNet50 (2015) [30]	25.6	88.03	98.44	82.44	97.01	83.22	97.25
ResNet101 (2015) [30]	44.5	88.51	98.44	84.10	97.34	84.20	97.45
ResNet152 (2015) [30]	60	**88.70**	**98.59**	84.85	97.80	84.75	97.58
ResNext50-32 (2016) [31]	25	87.95	98.44	81.17	96.67	84.81	97.65
Wide ResNet-50 (2016) [32]	69	88.21	98.59	82.20	97.28	85.27	97.81
DenseNet-121 (2017) [33]	8	86.95	98.26	83.03	97.14	82.45	96.93
EfficientNet-b4 (2019) [34]	19	81.28	97.37	**87.47**	**98.04**	**87.75**	**98.01**

**Table 4:** Ten CAFD classes best and worst detected by the ResNet152 model.

Best Detected CAFD Classes				Worst Detected CAFD Classes			
**Class**	**Precision**	**Recall**	**F1-Score**	**Class**	**Precision**	**Recall**	**F1-Score**
Sushki	0.96	1	0.98	Shashlik chicken with vegetables	0.71	0.67	0.69
Achichuk	0.95	1	0.98	Shashlik beef with vegetables	0.66	0.72	0.69
Sheep head	0.94	1	0.97	Shashlik chicken	0.67	0.74	0.7
Naryn	0.96	0.98	0.97	Shashlik minced meat	0.79	0.64	0.71
Plov	0.93	0.99	0.96	Asip	0.85	0.62	0.72
Tushpara with soup	0.93	0.97	0.95	Shashlik beef	0.74	0.69	0.72
Sorpa	0.97	0.93	0.95	Lagman without soup	0.83	0.68	0.75
Samsa	0.94	0.96	0.95	Kazy-karta	0.83	0.74	0.78
Hvorost	0.98	0.91	0.95	Beshbarmak with kazy	0.78	0.8	0.79
Manty	0.92	0.95	0.94	Tushpara fried	0.88	0.76	0.81

**Table 5:** Ten CAFD and Food1K classes best and worst detected by the EfficientNet-b4 model.

Best Detected CAFD and Food1K Classes				Worst Detected CAFD and Food1K Classes			
**Class**	**Precision**	**Recall**	**F1-Score**	**Class**	**Precision**	**Recall**	**F1-Score**
Sushki	0.91	1	0.96	Lagman without soup	0.6	0.27	0.37
Achichuk	1	0.95	0.97	Asip	0.88	0.38	0.53
Sheed head	0.94	0.94	0.94	Talkan-zhent	0.86	0.53	0.66
Airan-katyk	0.83	0.93	0.88	Doner lavash	0.75	0.6	0.67
Plov	0.97	0.90	0.93	Shashlik chicken with vegetables	0.88	0.64	0.74
Cheburek	0.92	0.90	0.91	Lagman fried	0.96	0.68	0.8
Irimshik	0.93	0.88	0.91	Doner nan	1	0.68	0.81
Samsa	0.93	0.88	0.90	Shashlik chicken	0.61	0.69	0.65
Naryn	0.97	0.87	0.92	Shashlik beef	0.67	0.69	0.68
Chak-chak	0.9	0.87	0.92	Kazy-karta	0.8	0.7	0.74

## Data Availability

The dataset, source codes, and pre-trained models are available open-source under MIT license in our GitHub https://github.com/IS2AI/Central-Asian-Food-Dataset (accessed on 20 March 2022) repository.

## Abbreviations

The following abbreviations are used in this manuscript:

AI	artificial intelligence
CAFD	Central Asian Food Dataset
CNN	convolutional neural network
CV	computer vision
ICCV	International Conference on Computer Vision
ML	machine learning
ResNet	residual network
